# Erythropoietin for Optic Neuritis

**DOI:** 10.18502/jovr.v14i3.4778

**Published:** 2019-07-18

**Authors:** Rod Foroozan

**Affiliations:** Division of Neuro-Ophthalmology, Baylor College of Medicine, Houston, TX, USA

Inflammatory optic neuropathy remains the most common acute optic neuropathy in patients under the age of 50 years. The most common cause for inflammatory optic neuropathy is sterile inflammation from demyelination, with multiple sclerosis (MS) as the most common associated condition. This is the type of optic neuropathy most commonly referred to as “optic neuritis.” The inflammation more commonly occurs posteriorly, does not cause optic disc edema, and is termed retrobulbar optic neuritis.

For the last 25 years or so, and primarily based on information from the optic neuritis treatment trial (ONTT),^[1]^ the standard treatment for acute inflammatory optic neuritis has been high dose corticosteroids, typically given intravenously for 3–5 days, followed by oral steroids. The expectation is that corticosteroids will hasten visual improvement without altering the degree of recovery of visual function. There has been a relative paucity of adjunctive agents that have been used in conjunction with corticosteroids. Given the persistent deficits that remain after a bout of optic neuritis, there remains an unmet need for neuroprotective therapy.

Erythropoietin, best recognized as a regulator of erythropoiesis, has shown potential neuroprotective properties in animal models of brain injury, including that from ischemia, trauma,^[2,3]^ epilepsy, and optic neuropathy.^[4,5]^ A rat model using immunization with myelin oligodendrocyte glycoprotein (MOG) showed functional and histopathological improvement of retinal ganglion cells and optic nerves when erythropoietin treatment was combined with high dose methylprednisolone therapy.^[6]^

The use of erythropoietin in humans with optic neuropathy has been reported in multiple studies from Iran. The treated conditions included traumatic optic neuropathy,^[7,8]^ nonarteritic anterior ischemic optic neuropathy,^[9,10]^ multiple sclerosis,^[11]^ and methanol optic neuropathy.^[12,13,14]^ With some exceptions (particularly methanol optic neuropathy), the proof of benefit has been rather underwhelming thus far; however, the balance of evidence suggests that there has been no harm to visual function (although one patient was reported to develop cerebral venous sinus thrombosis^[11]^).

One confounding issue with prior studies assessing the effect of erythropoietin on optic neuropathy is that the method of administration of the agent varied and included intravenous injection, intravitreal injection, and the use of microspheres. In addition, the dose of erythropoietin was inconsistent between the studies.

In this issue, Soltan Sanjari and coworkers assessed the addition of intravenous recombinant human erythropoietin (IVE) to intravenous methylprednisolone (IVMP) in patients with retrobulbar optic neuritis. Thirty-five patients were treated with IVMP alone and twenty-seven with the combination of IVMP and IVE (20,000 international units). Both groups were treated with intravenous agents for 3 days and were then given 11 days of oral prednisolone at 1 mg/kg. The primary outcome measure was the change in best corrected visual acuity, which was assessed up to 120 days. Other measures of visual function included contrast sensitivity (Yang vision tester), color vision (Ishihara pseudoisochromatic color plates), and automated perimetry (Humphrey 24-2 SITA Standard).^[15]^

While the cited inclusion criteria were described as those from the ONTT, it was not clear what ancillary laboratory studies were performed, and specifically whether blood was sent for aquaporin-4 and anti-MOG antibodies.^[15]^

By three months, there was no difference in the visual acuity between the two groups. In addition, there was no suggestion that the rate of visual recovery was different between the two groups. The mean deviation on automated perimetry showed no difference between the two groups at three months. There was also no difference in the contrast sensitivity and color vision between the two groups after treatment.^[15]^

The authors should be commended for enrolling a relatively large number of patients with inflammatory optic neuropathy within a short (10 day) window of symptoms. It was slightly disappointing that there were no structural, objective measures such as optical coherence tomography (OCT) of the retinal nerve fiber layer (RNFL), ganglion cell layer, or macula, particularly given the citation of work from another group which showed less RNFL thinning in patients with optic neuritis treated with IVE (33,000 international units) and IVMP for three days.^[16]^

While this was a negative study in terms of primary visual outcome, the authors point out that there was no evidence of clinically relevant side effects of IVE treatment. This is consistent with the prior literature. Regardless of the route of administration (including intravitreal injection), the use of erythropoietin appears to be safe, which is encouraging given the approval of a randomized, placebo-controlled trial [clinicaltrials.gov (NCT01962571)] from a group of investigators in Germany.^[17]^

So far, IVE for optic neuritis and demyelination^[18]^ has not proven to be a game changer. If additional studies are performed, I would encourage the authors to include additional measures of structure and function of the visual pathways to ensure that the focus is not solely on central visual function.

This is an open access journal, and articles are distributed under the terms of the Creative Commons Attribution-NonCommercial-ShareAlike 4.0 License, which allows others to remix, tweak, and build upon the work non-commercially, as long as appropriate credit is given and the new creations are licensed under the identical terms.
